# Elevated levels of serum urokinase plasminogen activator predict poor prognosis in hepatocellular carcinoma after resection

**DOI:** 10.1186/s12885-019-6397-3

**Published:** 2019-12-02

**Authors:** Ming-Chao Tsai, Yi-Hao Yen, Kuo-Chin Chang, Chao-Hung Hung, Chien-Hung Chen, Ming-Tsung Lin, Tsung-Hui Hu

**Affiliations:** 1 Division of Hepato-Gastroenterology, Department of Internal Medicine, Kaohsiung Chang Gung Memorial Hospital and Chang Gung University College of Medicine, 123 Ta Pei Road, Niao Sung Dist., Kaohsiung City, 83301 Taiwan; 2 Institute of Clinical Medical Sciences, Chang Gung University, Kaohsiung, 83301 Taiwan; 3 Division of Hepato-Gastroenterology, Department of Internal Medicine, Chiayi Chang Gung Memorial Hospital, Chiayi, 61363 Taiwan

**Keywords:** uPA, Hepatocellular carcinoma, Liver resection

## Abstract

**Background:**

Urokinase plasminogen activator (uPA) is an extracellular matrix-degrading protease that is involved in the invasiveness and progression of cancer. There is good evidence that uPA expression is a clinically relevant biomarker in some solid tumors, but its role in hepatocellulcar carcinoma (HCC) is uncertain. We evaluated the prognostic value of serum uPA before surgery in HCC patients receiving curative resection.

**Methods:**

Serum uPA levels were determined by enzyme-linked immunosorbent assay in 282 HCC patients who received complete liver resections at Kaohsiung Chang Gung Memorial Hospital. Overall survival (OS) curves were constructed using the Kaplan-Meier method and compared using the log-rank test. A Cox proportional -hazards regression model was used to identify independent prognostic factors. The median follow-up time was 52 months.

**Results:**

Patients with higher pretreatment serum uPA (≥1 ng/ml) had significantly shorter OS (*p* = 0.002). Patients with liver cirrhosis, hypoalbuminemia, and thrombocytopenia were significantly more likely to present with elevated uPA levels. Multivariate Cox regression analyses indicated that high pretreatment serum uPA [hazard ratio (HR), 1.848, *p* = 0.006], vascular invasion (HR, 2.940, *p* < 0.001), and pathology stage III/IV (HR, 3.517, *p* < 0.001) were independent prognostic factors for OS. In further stratified analyses, the combination of serum uPA and AFP had more capacity to predict OS.

**Conclusions:**

We conclude that uPA is a clinically relevant biomarker in HCC patients receiving curative resection, with higher expression of uPA being associated with higher mortality. This also highlights the potential utility of uPA as a therapeutic target for improved treatment strategies.

## Background

Hepatocellular carcinoma (HCC) is ranked as the second most common cause of cancer-related death worldwide and the fifth most frequent malignancy according to global cancer statistics [1, 2]. It is often considered to be linked to multiple risk factors, such as infections with hepatitis B virus (HBV) and hepatitis C virus (HCV), metabolic syndrome, and alcohol abuse [3]. The incidence of HCC has increased over the past decade, and it typically arises in the setting of liver cirrhosis. There is a wide variety of therapeutic options for patients with HCC, depending on liver function, performance status, and tumor burden [4]. To date, curative hepatic resection remains the most effective treatment for patients with HCC, especially in countries with a scarcity of donor organs [1]. However, the overall survival (OS) remains unsatisfactory.

Recent studies have shown that several parameters are independent predictors of survival after curative resection, such as tumor size, vascular invasion, resection margin status, and pathology stage [5]. Most of these factors are determined only after surgery and are not satisfactory in clinical practice for the prediction of outcomes. As a result, it is desirable to obtain simple serum biochemical markers that can be easily obtained at outpatient clinics before surgery to improve prognosis by allowing earlier intervention.

The urokinase-plasminogen activator (uPA) system comprises uPA, its receptor uPAR, and two inhibitors, PAI-1 and PAI-2. The system has a defined role in tissue degradation and extravascular fibrinolysis, and it is responsible for most of the activated plasminogen associated with cancer invasion and metastasis [6]. Furthermore, uPA is a member of the plasminogen activator system and a key serine protease that is involved in the conversion of inactive plasminogen into active plasmin, which in turn functions in a range of events of the metastatic cascade [7]. Many studies have shown that uPA overexpression is associated with a worse prognosis in many cancers, including breast cancer, lung cancer, and ovarian cancer [8–11]. However, only one study has been conducted for HCC patients so far [12]. Zheng et al. showed that the concomitant overexpression of uPA and its receptor uPAR correlate with HCC invasiveness and metastasis.

As uPA can be shed from tumor stroma into the blood vessels, the potential use of circulating uPA in serum has been first explored in the late 1980s. Since then, many studies have found higher serum uPA levels in cancer patients than in healthy individuals [13]. However, there have been no studies that have investigated serum uPA in HCC patients. Hence, we conducted this study to test the hypothesis that elevated serum uPA in pretreatment serum samples predict poor prognosis in HCC patients undergoing curative resection.

## Methods

### Patients and follow-up

This retrospective study included 287 patients who underwent curative hepatic resection for HCC at Kaohsiung Chang Gung Memorial Hospital between January 2006 and April 2015. HCC was diagnosed histologically based on tumor resection. All patients were followed up in the outpatient clinic with regular surveillance for recurrence by serum AFPs level and ultrasound at week 4, and every 12 weeks afterwards. Abdominal computed tomography (CT) or magnetic resonance image (MRI) were performed at the 1st month after liver section and every 12 months or recurrence is suspected clinically. The demographics, clinical characteristics, and pathological findings of HCC were recorded. Disease-free survival (DFS) was defined as the period from tumor removal by resection until the detection of recurrent or metastatic disease according to liver CT or MRI studies. OS was measured from the date of surgery until the date of death, the last observation or December 312,017.

This study was approved by the Institutional Review Broad of Chang Gung Memorial Hospital (No. 201901103B0) and was conducted in accordance with the Declaration of Helsinki and current ethical guidelines. Written informed consent was obtained from all patients prior to surgery.

### Sample collection and assay of serum uPA level

Serum samples were collected from TissueBank and BioBank at Kaohsiung Chang Gung Memorial Hospital. The samples had been collected before the hepatic resection and were stored at − 80 °C. The serum levels of uPA were assessed using independent sandwich enzyme-linked immunosorbent assay (ELISA) kits (GB BioFibroScore® FibA kits, General Biologicals Corporation, Hsinchu, Taiwan). The analytes were recognized by antibodies coated on the ELISA plate and horseradish peroxidase (HRP)-conjugated 2nd antibody.

Stop solution was added to each well, and the optic density (OD) value (450 nm) was measured by an ELISA reader (Emax, Molecular Devices, Sunnyvale, CA, USA). The serum level of each specimen was calculated by interpolation with quantitative standards. A total of 120 μL of archived serum sample was thawed to assess the uPA. The sensitivity of the assay for uPA was 10 μg/L, and the intra-assay and inter-assay coefficient variations of the uPA ELISA kit were < 3.6 and < 3.7%, respectively .

### Statistical analysis

Statistical analyses were performed using SPSS software version 21 (Chicago, IL, USA). The experimental values of continuous variables are expressed as the mean ± standard error of the mean. The chi-squared test was used as appropriate to evaluate the significance of differences in data between groups. DFS and OS were determined using the Kaplan-Meier method, and comparisons were made using the log-rank test .

Independent risk factors for DFS and OS were identified through a Cox regression analysis. Potential risk factors with a *P*-value < 0.3 in the univariate analysis were included in the multivariate analysis. The area under the receiver operating characteristic curve (AUROC) was used to estimate the predictive accuracy of uPA and AFP. The uPA value with the highest Youden’s index (sensitivity + specificity − 1) yielded by the ROC analysis of diagnostic accuracies for OS was selected as the best cutoff value. A *p* value of < 0.05 was considered statistically significant.

## Results

### Patient characteristics

The clinical-pathological data of the 287 HCC patients who underwent curative resection are summarized in Table 1. There were 232 males (80.8%) and 55 females (19.2%), and the mean age was 60 years (range: 29–84 years). The etiology for HCC was hepatitis B virus (HBV) in 140 patients, hepatitis C virus (HCV) in 85 patients, coinfection with both hepatitis viruses in 21 patients, and unknown in 41 patients. The mean diameter of largest tumor was 4.6 cm (range 1–9.5 cm). Pathological findings revealed vascular invasion (microvessel or macrovessel invasion) in 160 patients. The mean follow-up time was 52 months (range 1–83.8 months). Recurrence occurred in 142 patients (49.5%), whereas 87 patients (30.3%) died during follow-up.

**Table 1:** Clinicopathological features of 287 HCC patients undergoing curative resection

Patient demographics	
Age (years)	59.6 ± 11.7
Sex (M: F)	232: 55
AFP (ng/mL)	6122.1 ± 42,202.5
Albumin (mg/dl)	3.4 ± 0.6
Total bilirubin (mg/dl)	1.0 ± 2.4
uPA (ng/ml)	1.0 ± 1.4
Tumor size (cm)	4.6 ± 3.5
Liver cirrhosis, n (%)	123 (42.9%)
Hepatitis (B: C: B + C: NBNC)	140: 85: 21: 41
Pathological features	
Vascular invasion (Yes: No)	160: 127
Tumor differentiation (well: moderate: poor)	38: 236: 12
Histological grade (I: II: III: IV)	105: 129: 49: 4

uPA, urokinase-type plasminogen activator

### ROC curves of serum uPA and AFP for HCC OS

**Fig. 1:**
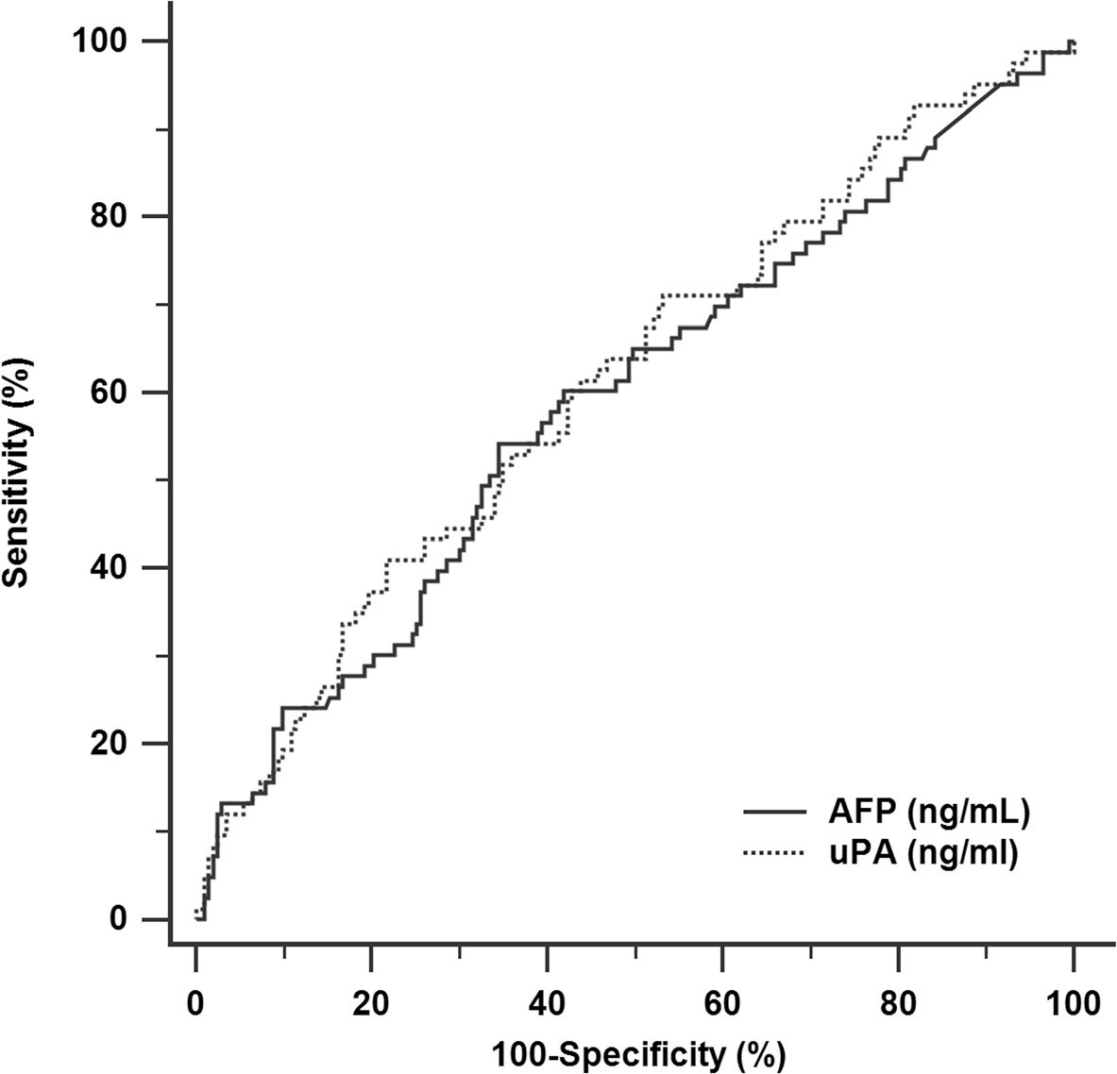
Receiver operating characteristic (ROC) curves for serum AFP and uPA in relations to mortality

Levels of serum uPA were measured in 287 patients, and the median concentration was 0.7 ng/ml (mean 1.0 ng/ml, range 0.2–14.7 ng/ml, standard deviation 1.36 ng/ml). The ROC curves for serum uPA and AFP markers in relation to overall survival are shown in Fig. 1. Each marker was stratified according the maximum sensitivity and specificity using Youden’s index. The optimal cutoff value for uPA was 1.005 (AUROC curve: 0.611; 95% confidence interval (CI): 0.538–0.683, *p* = 0.003). The optimal cutoff value for AFP was 201 (AUROC curve: 0.590; 95% CI: 0.516–0.664, *p* = 0.017). The predictive accuracy of uPA for overall survival is greater than AFP; however, the superiority was not statistically significant.

**Fig. 2:**
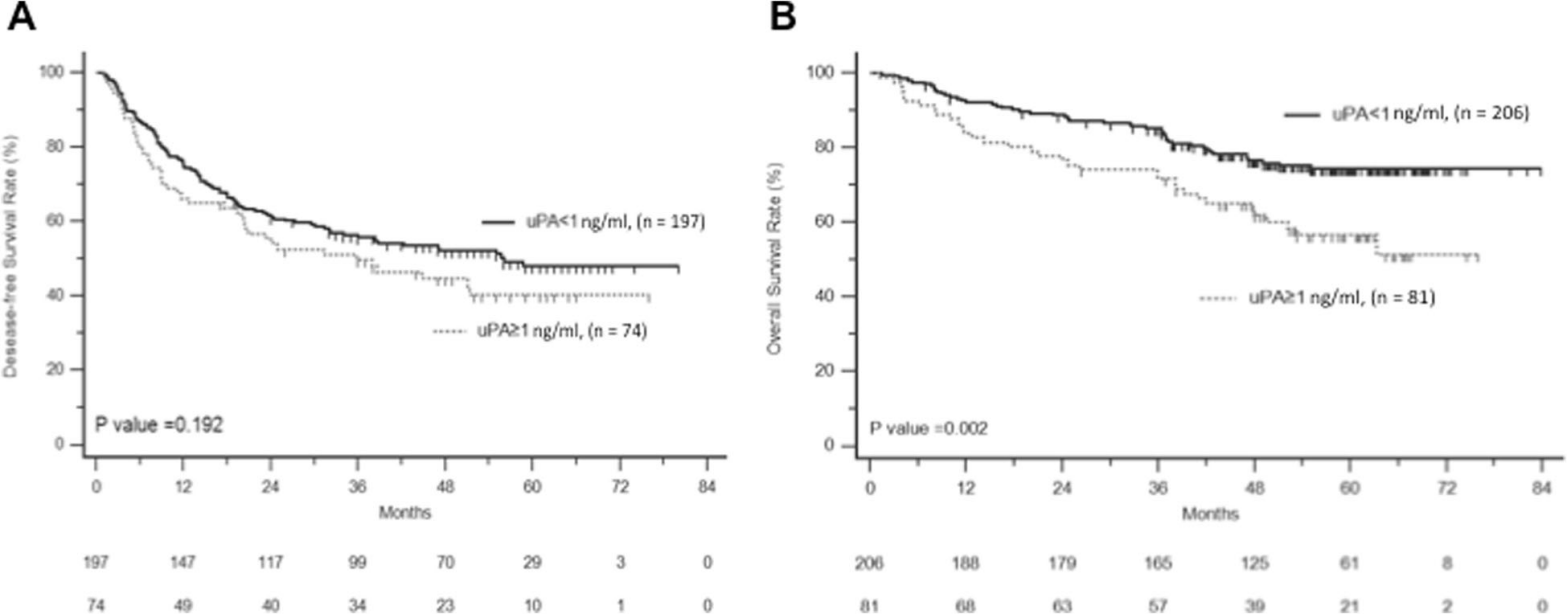
Disease-free survival (**a**) and overall survival (**b**) of HCC patients after curative resection stratified by serum uPA

The optimal cut-off value of serum uPA was 1 ng/ml with a sensitivity of 41% and specificity of 77.5% for the prediction of death. When using this value, 28% of the patients had high uPA levels (≥1 ng/ml). The Kaplan-Meier estimates of DFS and OS stratified by serum uPA were dichotomized (≥1 ng/ml and < 1 ng/ml) and are shown in Fig. 2. Patients with high serum uPA had significantly shorter OS than those with low serum uPA (*p* = 0.002), but this was not in DFS (*p* = 0.192).

### Relationship between clinicopathological features and uPA levels

The clinicopathological features of HCC patients with different uPA status are summarized in Table 2. High serum uPA was associated with hypoalbuminemia (*p* < 0.001), thrombocytopenia (*p* = 0.032), and liver cirrhosis (*p* < 0.001), but not with other characteristics such as gender, age, etiology, AFP, tumor size, vascular invasion, and pathological stage.

**Table 2:** Association between serum uPA and clinical characteristics in 287 HCC patients undergoing curative resection

	High uPA (*n* = 81)	Low uPA (*n* = 206)	*P*-value
Age (years)	60.5 ± 12.9	59.2 ± 11.2	0.431
Male gender, n (%)	61 (75.3%)	171 (83.0%)	0.136
Total bilirubin (mg/dl)	0.9 ± 0.7	1.0 ± 2.8	0.804
Albumin (mg/dl)	3.2 ± 0.5	3.5 ± 0.6	< 0.001
Platelet < 150 × 10^9^/L, n (%)	46 (56.8%)	88 (42.7%)	0.032
AFP > 200 ng/mL, n (%)	24 (29.6%)	42 (20.4%)	0.094
Liver cirrhosis, n (%)	48 (59.3%)	75 (36.4%)	< 0.001
Tumor size > 5 cm, n (%)	24 (29.6%)	64 (31.1%)	0.812
Vascular invasion, n (%)	46 (56.8%)	114 (55.3%)	0.824
Pathology stage III + IV, n (%)	19 (23.5%)	34 (16.5%)	0.172

High uPA: uPA ≥1 ng/ml; low uPA: uPA < 1 ng/ml

### Univariate and multivariate analyses of independent risk factors

There were 87 patients (30.3%) who died after a mean follow-up time of 52 months. The univariate analyses demonstrated that AFP ≥ 200 ng/ml (hazard ratio [HR], 2.012; 95% CI, 1.088–3.143; *p* = 0.002), uPA ≥ 1 ng/ml (HR, 1.968; 95% CI, 1.271–3.049; *p* = 0.002), tumor size > 5 cm (HR, 2.402; 95% CI, 1.515–3.663; *p* < 0.001), vascular invasion (HR, 3.812; 95% CI, 2.268–6.407; *p* < 0.001), and pathology stage (III/IV vs. I/II) (HR, 4.980; 95% CI, 3.226–7.687; *p* < 0.001) were associated with OS. All significant covariates in the univariate analyses were entered into a multivariate Cox analysis. Serum uPA (HR, 1.848; 95% CI, 1.191–2.869; *p* = 0.006), vascular invasion (HR, 2.914; 95% CI, 1.640–5.178; *p* < 0.001), and pathology stage (HR, 3.546; 95% CI, 2.227–5.648; *p* < 0.001) emerged as independent prognostic factors for OS (Table 3).

**Table 3:** Univariate and multivariate analysis of prognostics factors for overall survival in HCC patients after curative resection

		Univariate		Multivariate	
Variable	Comparison	HR (95%CI)	*P* value	HR (95%CI)	*P* value
Age (years)	≥65 vs. < 65	1.311 (0.857–2.006)	0.212		
Gender	Male vs. Female	0.990 (0.583–1.682)	0.971		
Total bilirubin (mg/dl)	Per 1 unit increase	0.986 (0.888–1.095)	0.787		
Albumin (mg/dl)	Per 1 unit increase	1.001 (0.692–1.447)	0.998		
Platelet (× 10^9^/L)	< 150 vs. ≥150	1.028 (0.675–1.566)	0.897		
AFP (ng/mL)	≥200 vs. < 200	2.012 (1.288–3.143)	0.002		
uPA (ng/ml)	≥1 vs. < 1	1.968 (1.271–3.049)	0.002	1.848 (1.191–2.867)	0.006
Liver cirrhosis	Yes vs. No	1.039 (0.680–1.588)	0.859		
Tumor size (cm)	≥5 vs. < 5	2.402 (1.575–3.663)	< 0.001		
Vascular invasion	Yes vs. No	3.812 (2.268–6.407)	< 0.001	2.940 (1.655–5.224)	< 0.001
Pathology stage	III + IV vs. I + II	4.980 (3.226–7.687)	< 0.001	3.517 (2.208–5.600)	< 0.001

### Prognostic value of serum uPA based on AFP levels

**Fig. 3:**
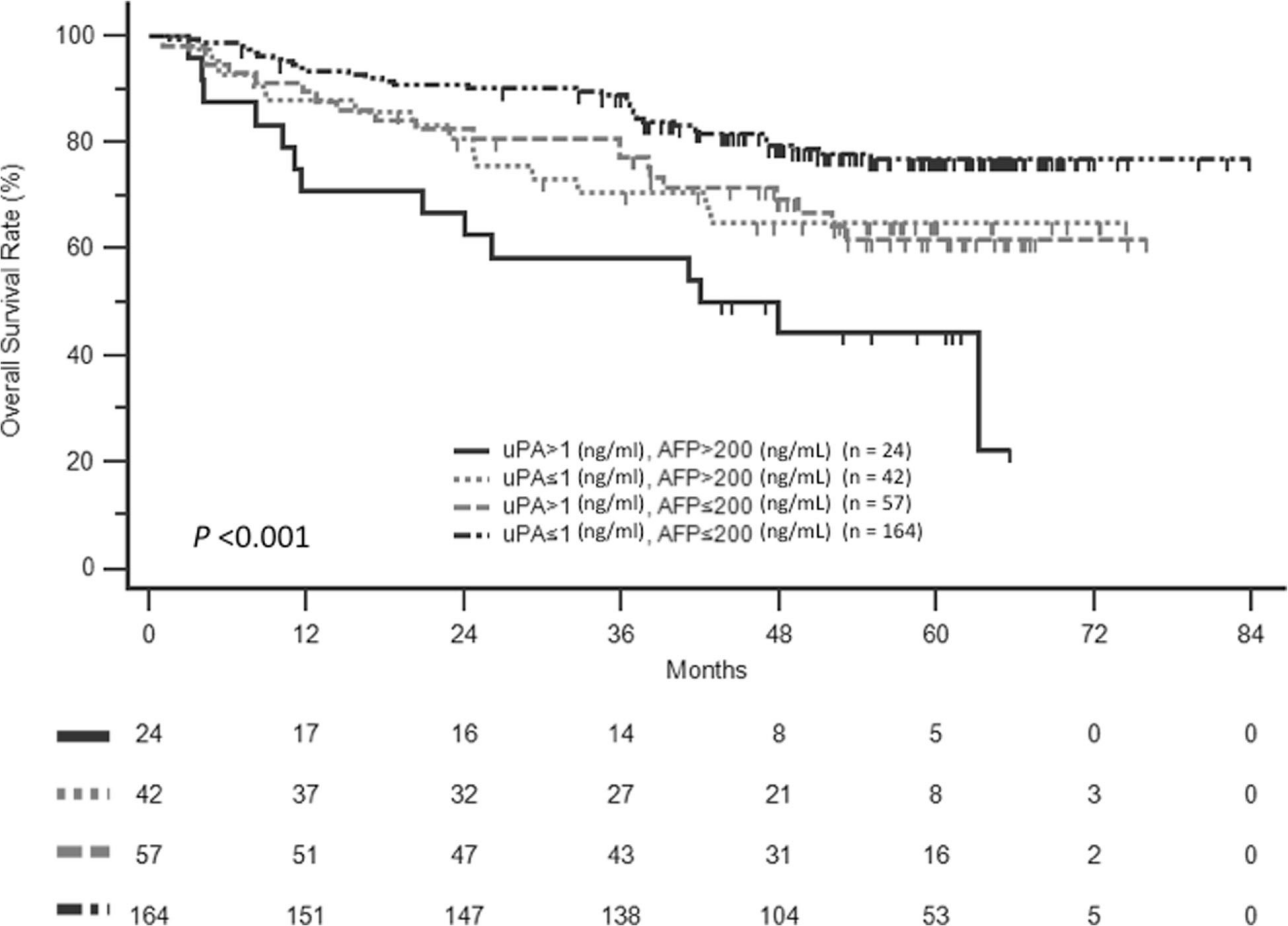
Overall survival of HCC patients after curative resection stratified by the combination of serum uPA and AFP levels

Since the univariate analysis indicated that preoperative AFP ≥200 ng/ml was a predictor of poor OS, we examined whether the prognostic value of serum uPA varied with the AFP level. When serum uPA and AFP were considered together, the patients were divided into four groups based on the following: uPA ≥1 ng/ml and AFP ≥200 ng/ml (*n* = 24); uPA < 1 ng/ml and AFP ≥200 ng/ml (*n* = 42); uPA ≥1 ng/ml and AFP < 200 ng/ml (*n* = 57); and uPA < 1 ng/ml and AFP < 200 ng/ml (*n* = 164). Figure 3 shows that the OS rates were significantly higher in patients with uPA < 1 ng/ml and AFP < 200 ng/ml compared with other groups (*p* < 0.001).

## Discussion

This is the first study of serum uPA levels in HCC patients. We found that HCC patients with high pre-operative uPA (serum uPA ≥1 ng/ml) exhibited lower OS rates after curative hepatic resection surgery. Furthermore, the combination of serum uPA and AFP could also be important in determining postoperative outcomes in response to the surgical procedure. These results may not only assist surgeons in predicting HCC patient survival but remind clinical physicians to perform timely adjuvant treatments to improve the prognosis of patients with high preoperative serum levels of uPA.

Many studies have investigated the clinical impact of the expression of members of the uPA system and their correlation with prognosis in a wide variety of cancers [8]. However, only one study has been conducted for HCC patients so far [12]. In 2000, Zheng et al. found that increasing uPA protein levels in HCC tissue was associated with increased invasion and metastasis in 22 HCC patients [12]. In order to explore a possible correlation of uPA between HCC and paired non-HCC tissues, we analyzed TCGA datasets, which was established by NCI/NIH and publicly available (https://tcga-data.nci.nih.gov/tcga/). Our analysis found that the level of uPA is significant higher in HCC tissues compared with their paired non-tumor tissues (data not shown). Nevertheless, commercially available tests are not extensively used because they require a great amount of fresh-frozen tissue or formalin-fixed paraffin-embedded samples. Aside from detection in the tissue, uPA can be shed by stromal cells or tumor cells into the bloodstream and measured in serum or plasma. Many studies have indicated that serum uPA can be used to predict the outcomes of cancer patients, although conflicting results have been reported, and none of the studies have been conducted with HCC patients [14–18]. In the current study, we found that elevated serum uPA levels were related to poorer OS in HCC patients undergoing resection, regardless of whether it is evaluated dichotomously (*p* = 0.002) or continuously (*p* = 0.005). The multivariate Cox regression analysis indicated that elevated serum uPA was an independent prognostic factor for OS in HCC patients (*p* = 0.006). These results are consistent with those obtained with other cancers, including prostate cancer [19] and gastrointestinal cancer [20]. Recently, Wei et al. showed that SPINK13, a suppressor of the proliferation of HCC cells, directly interacted with uPA, inhibited the cleavage of MMP9 by uPA, and achieved antitumor activity on HCC cells [21]. Future clinical studies are required to verify whether high serum uPA might be useful for identifying HCC patients who are most likely to benefit from SPINK13, and trials for adjuvant treatment after resection are being planned.

AFP is the most useful and cost-effect serum biomarker to evaluate the prognosis of AFP-positive HCC patients in clinical practice. However, in the present study, AFP was only significant in univariate but not in multivariate analysis for OS prediction. This result is different from the previous studies [22, 23], in which the AFP level at diagnosis was an independent risk predictor associated with overall survival. However, some studies found the preoperative AFP level does not correlated with postoperative survival [24, 25], which is consistent with the present result. The reasons might be due to the sample size and the heterogeneity of tumors and therapies with curative intent. In the present study, we found that the AFP level was associated with tumor differentiation, and tumor size. In multivariate analysis, if we removed the factor of pathologic grade, the AFP is an independent risk factor associated with poor OS. The condition is similar with tumor size. However, in the study by Bai et al. [22], they did not put the tumor pathologic grade into multivariate analysis, which might affect the power of prediction of AFP. To consider the impact of serum AFP on OS as much as possible, serum uPA and AFP were considered together. We found that the OS rates were significantly poorer in those with high uPA and AFP than in other groups. This demonstrated that the combination of serum uPA and AFP had more capacity to predict patients’ outcomes. Importantly, in subgroups with low or high AFP levels, serum uPA still had the ability to discriminate HCC patients undergoing curative resection with good prognoses from those with poor OS. More studies are required to validate the clinical role in different types of management, such as radiofrequency ablation (RFA) or transcatheter arterial chemoembolization (TACE).

We found that serum uPA was significantly elevated in HCC patients with liver cirrhosis, hypoalbuminemia, and thrombocytopenia, which provides additional evidence that elevated serum uPA may play a role in the prediction of liver fibrosis severity. Hepatic fibrosis is characterized by the progressive deposition of extracellular matrix (ECM) in patients with chronic liver injury. Hepatic stellate cells (HSCs) are the major source of ECM in the liver. They are also the primary cellular mediator of hepatic fibrosis through their transdifferentiation, or activation from a vitamin A-storing cell to a contractile, matrix-producing myofibroblast in response to liver injury and inflammation [26, 27]. TGF-β triggers the uPA pathway, which activates quiescent HSCs and causes ECM deposition [28]. Hence, uPA is considered to be involved in liver fibrosis through the regulation of HSCs. Actually, uPA is thought to be a particular serine protease, which directly degrades ECM and catalyzes the activation of latent matrix metalloproteinases (MMPs) [29]. In the CCl_4_-induced acute liver injury model, lack of uPA led to the accumulation of fibrin and excessive matrix [30]. Hence, the anti-fibrotic activity of uPA was confirmed in animal models of liver fibrosis. Theoretically, the severity of liver fibrosis should be negatively correlated with serum uPA levels. However, in the present study, serum uPA was associated with the fibrosis stage in this study (*p* < 0.001, Additional file 1: Figure S1). This result is consistent with a recent study by Liu et al., in which indicated that the stage of hepatic fibrosis in HCV-infected patients is positively correlated with serum uPA levels [31]. We supposed that in acute liver injury phase, the uPA can protect liver to avoid fibrin and matrix accumulation. In chronic liver fibrotic phase, increasing uPA expression in fibrotic liver tissues may reverse fibrosis and regenerate functional hepatocytes. However, we need more studies to support our view here.

Aside from uPA, our univariate analyses showed that the AFP level, tumor size, vascular invasion, and pathology stage were also significant prognostic factors associated with the OS of HCC patients undergoing curative resection. However, the multivariate analysis demonstrated that only vascular invasion and the pathology stage were independent prognostic factors for OS. This is consistent with the results of previous studies in which tumor-related factors (vascular invasion and histological stage) determined the outcomes of HCC patients undergoing resection [32].

Our study had three main limitations. First, only HCC patients undergoing resection were enrolled. Second, this is a retrospective study, and some patients were lost to follow-up after operation. Third, all patients in this cohort were treated at a tertiary medical center, which means that referral bias could not be completely avoided. These data should be validated externally in other regions of the world. Finally, more information from patients with chronic liver disease without HCC, as well as healthy individuals, should be collected for further investigation together, which will be included in our future work.

## Conclusions

In summary, we have presented for the first time that the serum uPA level is a clinically relevant biomarker in HCC patients receiving curative resection, with higher expression of uPA being associated with higher mortality. This also highlights the potential utility of uPA as a therapeutic target for improved treatment strategies. A recent study on a uPA inhibitor, SPINK13, provided exciting and promising evidence of an antitumor effect in a mouse model of HCC. Thus, future research should aim at clarifying whether elevated serum uPA may be useful for identifying patients that are likely to derive clinical benefit from a targeted strategy.

## Supplementary information

**Additional file 1: Figure S1.** Correlations between serum uPA and fibrosis score in HCC patients undergoing curative resection.

## Data Availability

The datasets used and/or analyzed during the current study are available from the corresponding author on reasonable request.

## Abbreviations

HCC: Hepatocellular carcinoma
uPA: Urokinase plasminogen activator
