# Supplementary figures and images for: Elevated levels of serum urokinase plasminogen activator predict poor prognosis in hepatocellular carcinoma after resection
Source: BMC Cancer. 2019 Dec 2;19:1169. doi: 10.1186/s12885-019-6397-3 (PMC6889356; doi:10.1186/s12885-019-6397-3)

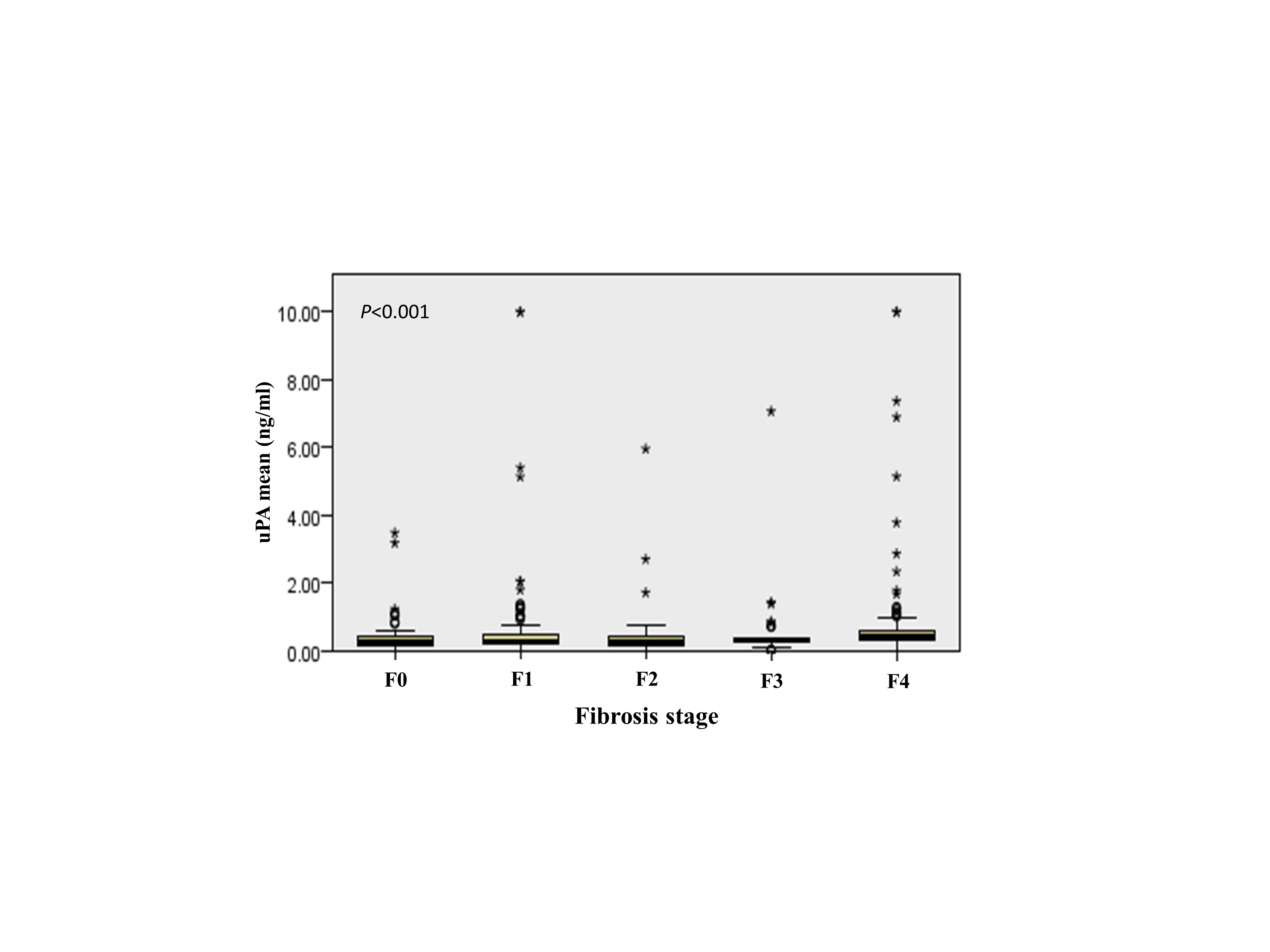

Supplement: Supplementary file 1 — Additional file 1: Figure S1. Correlations between serum uPA and fibrosis score in HCC patients undergoing curative resection. [file 12885_2019_6397_MOESM1_ESM.tif]
